# Oxidative modifications, mitochondrial dysfunction, and impaired protein degradation in Parkinson's disease: how neurons are lost in the Bermuda triangle

**DOI:** 10.1186/1750-1326-4-24

**Published:** 2009-06-05

**Authors:** Kristen A Malkus, Elpida Tsika, Harry Ischiropoulos

**Affiliations:** 1 The Joseph Stokes Jr Research Institute, The Children's Hospital of Philadelphia, Philadelphia, Pennsylvania, USA; 2 The Department of Molecular Biology and Genetics, Democritus University of Thrace, Alexandroupolis, Greece; 3 Department of Pediatrics, The University of Pennsylvania, Philadelphia, Pennsylvania, USA; 4 Department of Pharmacology, The University of Pennsylvania, Philadelphia, Pennsylvania, USA

## Abstract

While numerous hypotheses have been proposed to explain the molecular mechanisms underlying the pathogenesis of neurodegenerative diseases, the theory of oxidative stress has received considerable support. Although many correlations have been established and encouraging evidence has been obtained, conclusive proof of causation for the oxidative stress hypothesis is lacking and potential cures have not emerged. Therefore it is likely that other factors, possibly in coordination with oxidative stress, contribute to neuron death. Using Parkinson's disease (PD) as the paradigm, this review explores the hypothesis that oxidative modifications, mitochondrial functional disruption, and impairment of protein degradation constitute three interrelated molecular pathways that execute neuron death. These intertwined events are the consequence of environmental exposure, genetic factors, and endogenous risks and constitute a "Bermuda triangle" that may be considered the underlying cause of neurodegenerative pathogenesis.

## Review

### A "Bermuda Triangle" of Insults Induces Neurodegeneration

Understanding the molecular basis of neurodegenerative diseases has proven to be a major challenge, yet is vitally important because of the prevalence of these chronic conditions in the aging population. While diverse neurodegenerative disorders, which encompass Alzheimer's disease (AD), Parkinson's disease (PD), Huntington's disease (HD), and Amyotrophic Lateral Sclerosis (ALS), involve unique proteins and selectively disparate brain regions, they share two key features: formation of insoluble protein aggregates and neuron degeneration [1]. Therefore it is reasonable to speculate that a common causative process underlies the pathogenesis of neurodegenerative disorders. Specifically focusing on PD, this review proposes that neurodegeneration is due to three interrelated molecular mechanisms: oxidative modifications, mitochondrial dysfunction, and impaired protein degradation.

One possible unifying molecular mechanism that can induce both the formation of protein inclusions and neuron degeneration is the oxidative reactions derived from the production of reactive oxygen and nitrogen species. A substantial increase in oxidized proteins, lipids, and DNA has been found in postmortem brain tissue of PD patients as compared to age-matched disease-free subjects [2]. Although these observations do not demonstrate that oxidative processes are the sole cause of neuronal demise, they are consistent with data in animal and cellular model systems (reviewed below) that establish a role for oxidation in neurodegeneration and death.

The four electron reduction of oxygen to water is a fundamental biochemical process largely responsible for the survival of organisms in aerobic environments. Oxidation and reduction reactions are also important in the central nervous system for the formation and metabolic processing of catecholamines, for the production of signaling molecules such as nitric oxide, and for the metabolism of xenobiotics. The coupling of these enzymatic systems ensures that electrons are transferred to the desired substrate, avoiding partial reduction of oxygen to reactive species. However, inappropriate reduction of oxygen does occur occasionally, resulting in the production of superoxide and hydrogen peroxide.

Mitochondria are considered a key source of reactive species. Interruptions or disturbances in the electron transport chain allow electrons to be transferred and reduce molecular oxygen by one electron to form superoxide, or by two electrons to form hydrogen peroxide. In addition to generating ATP, mitochondria also play critical roles in regulating cellular viability. Therefore, functional compromise of this organelle has a large impact on oxidative homeostasis. To protect against reactive species, a network of antioxidant enzymes including Cu, Zn superoxide dismutase (SOD) in the cytosol, Mn SOD in the mitochondria, peroxidases, and catalase secure conversion of these reactive species to water and therefore prevent adverse oxidation of cellular macromolecules.

How then do reactive species induce stress? The answer to this question is not entirely understood but several suggestions have been advanced. A significant increase in the rate of reactive species production, potentially coupled with a decline in the efficiency of the antioxidant networks that remove them may permit secondary reactions with cellular biomolecules (proteins, lipids, DNA/RNA) that result in undesired oxidations. While neuronal homeostasis can be disturbed by these oxidative modifications, protective mechanisms including protein degradation, lipid turnover, and DNA repair sustain cellular homeostasis by repairing or removing the oxidized macromolecules. However, compromise of these defense mechanisms – either by direct oxidative modification or indirectly by the inability to process oxidatively modified substrates – may render the cell incapable of efficiently removing oxidized biomolecules, resulting in their accumulation.

Alteration of protein folding and degradation, due to oxidative stress, mitochondrial dysfunction, or other factors has been strongly associated with neurodegenerative diseases. Protein aggregation is a hallmark of a diverse array of these late-onset neurodegenerative disorders, and thus factors that influence protein folding, processing, and clearance have been a focus of much research. Two major pathways are responsible for the degradation of cellular proteins: the ubiquitin-proteasome system (UPS) [3] and the autophagy-lysosome pathway [4-6].

The UPS is the principal mechanism of degradation for short-lived proteins and proteins that are misfolded in the endoplasmic reticulum [5]. UPS substrates are selectively targeted for degradation by the 20S or 26S proteasome complex after the conjugation of a polyubiquitin tag through a three-step enzymatic cascade [7]. Upon recruitment to the proteasome, the substrates must be unfolded to pass through the narrow barrel of the proteasome where they are degraded [5,7]. The consistent observation that antibodies against ubiquitin label some of the human and rodent protein inclusions suggests that failure of the UPS may contribute to neurodegeneration. However, the effect of UPS inhibition on cell death and protein aggregation in cellular model systems as well as rodent models has yielded conflicting results that have not been entirely resolved [8-13]. These variable results suggest that other factors, including other protein degradation pathways such as autophagy and mitochondrial dysfunction associated with a decline in ATP levels, may contribute to cellular viability. This hypothesis remains to be further explored in cellular and rodent model systems.

The other primary pathway for protein degradation in the cell is through autophagy. While the ultimate result of autophagy is always the delivery of proteins or organelles to the lysosome for degradation, there are three different routes by which this can be accomplished. Macroautophagy is a non-selective method of bulk degradation whose activity is upregulated in response to stress. Microautophagy is also a non-selective process, though it is maintained at a constitutively active state. The final type of autophagy is chaperone-mediated autophagy (CMA). Like macroautophagy, CMA is present at low basal levels in the cell and is upregulated in response to stress. However, CMA is unique from the other two forms of autophagy in that it is a selective process [4,14]. While the UPS, macroautophagy, and CMA have been implicated as potential contributors to neurodegeneration, their precise involvement is controversial and unclear.

Macroautophagy was first implicated in neurodegeneration after it was noted that autophagic structures were present in affected brain regions of patients with neurodegenerative diseases, including PD [15-18]. Initial hypotheses speculated that these autophagic vacuoles were evidence of neurons "eating themselves to death" [15]. This was based on previous observations that autophagic mechanisms can participate in induction of non-apoptotic cell death cascades [19-25]. However, recent evidence has shown that, particularly within the context of neurodegeneration, macroautophagy may instead serve as a protective process by which cells attempt to clear misfolded proteins and damaged organelles[4]. Independently generated data has revealed the neuroprotective role of macroautophagy through manipulation of either Atg7 or Atg5 – two different proteins essential for autophagy. Conditionally knocking out either of these genes in the central nervous system of mice leads to severe neurodegeneration and formation of protein inclusions, accompanied by motor dysfunction and early death [26,27]. In cells, inhibition of macroautophagy at the stage of autophagosome formation by 3-methyladenine (3-MA), at the stage of autophagosome-lysosome fusion by Bafilomycin A1 (BafA1), or at the stage of lysosomal degradation by deficiency of the enzyme cathepsin D, led to enhanced aggregation of polygluatmine, polyalanine, and α-synuclein proteins [28-30]. In contrast, the induction of autophagy leds to increased clearance and reduced toxicity of pathogenic proteins, decreased aggregate formation and neurodegeneration, and improved behavioral phenotype in fly and mouse models [29-35]. Stimulation of autophagy has been accomplished either by rapamycin, which inhibits the negative regulator of autophapgy mammalian target of rapamycin (mTOR), or by several mTOR independent compounds including lithium, trehalose, and small molecules identified in a screen [29-35].

CMA may also be playing a role in cell vulnerability. In CMA deficient cells, baseline levels of survival were unaffected, but stressors such as UV light or multiple types of oxidative stress significantly reduced viability [36]. Additionally, the proteins implicated in neurodegenerative disease, APP, Htt, and α-synuclein, all contain a putative CMA targeting motif, indicating that regulation of this degradation system may have important effects on pathogenic protein homeostasis[14].

The UPS, macroautophagy, and CMA are each involved in the degradation of oxidized proteins. In response to moderate levels of oxidative stress, cells are able to induce a protective upregulation of all three of these protein degradation pathways, supporting an interplay between protein oxidation and protein degradation during normal homeostasis [4,37-43].

However, more severe oxidative stress impairs the degradation of oxidized proteins [39,40,44]. For the UPS system, oxidative modifications that induce crosslinking, misfolding, and aggregation prevent the proper unfolding necessary for substrates to be passed through the barrel of the proteasome for degradation, making these substrates resistant to degradation as well as potentially inhibiting the overall activity of the proteasome [45-48]. Additionally, direct oxidative modification of the proteasome subunits inhibits 20S and 26S catalytic peptidase activity [46,49-54]. In a rat model of ischemia/reperfusion, the lipid peroxidation product 4-hydroxyl-2-noneal (HNE) impaired the peptidase activity of the proteasome by direct oxidative modification of the α-like 20S proteasome subunits iota, C3, and an isoform of XAPC7 [53,54].

Additionally, oxidatively modified proteins may impair the cellular machinery of autophagic degradation [55]. Reactive species can damage the lysosomal membrane and crosslink membrane proteins, resulting in cytosolic leakage of lysosomal hydrolases [56-58]. Some oxidatively modified aggregated species are resistant to degradation by proteases and accumulate within lysosomes. There, the non-degraded proteins become a potential new source of reactive species, further damaging the lysosomal membrane [59].

**Figure 1:**
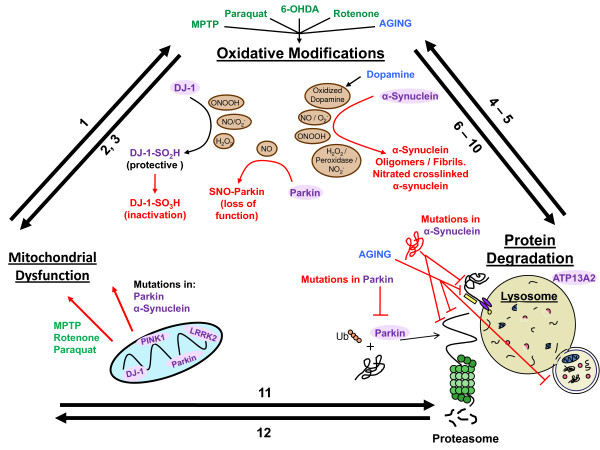
**A "Bermuda Triangle" of insults leads to neurondeath in PD**. Known risk factors for the onset of Parkinson's disease (PD) include environmental (green), genetic (purple), and endogenous (blue) influences. Contributions from these risk factors trigger oxidative modifications, mitochondrial dysfunction, and impaired protein degradation that together form a "Bermuda triangle" of interrelated molecular events that underlie neurodegeneration. The interactions between these pathways are supported by the following (for details and citations, please refer to text): (1) Disturbances in mitochondrial respiration generate reactive oxygen species. (2) Overexpression of SOD is protective against mitochondrial toxins. (3) NOS deficiency or inhibition attenuates MPTP, paraquat, and rotenone toxicity. (4) Inhibition of degradation systems leads to increased sensitivity to oxidative stressors. (5) Impaired degradation leads to an accumulation of substrates, increasing the probability for oxidative modifications. (6) Excessive production of reactive oxygen and nitrogen species modifies proteins, leading to inactivation, crosslinking, and aggregation. (7) α-Synuclein modified by oxidized dopamine impedes CMA. (8) Oxidative modifications modify the lysosomal membrane and crosslink membrane proteins. (9) UPS and CMA are not able to unfold and remove oxidatively proteins. (10) Oxidative modification of proteasome subunits inhibits UPS function. (11) Macroautophagy is the principle mechanism for the degradation of damaged mitochondria. (12) Proteasome inhibition increases mitochondrial reactive species generation and decreases complex I and II activity.

Below we discuss evidence that implicates known environmental, genetic, and endogenous factors as contributors that intitiate oxidative modifications, mitochondrial dysfunction, and protein aggregation in PD (Figure 1). We propose that the combined interactions of these three interrelated molecular pathways – oxidative modifications, mitochondrial dysfunction, and impaired protein degradation – constitute a "Bermuda Triangle" that ultimately induces neuron death.

### Environmental Toxins

One of the most striking clues into the processes involved in PD came from the observation of rapid-onset motor impairments that replicated most of the features of sporadic PD in individuals accidentally exposed to 1-Methyl-4-phenyl-1,2,3,6-tetrahydropyridine (MPTP) [60]. Further epidemiological studies have suggested that exposure to other pesticides and environmental toxins are associated with PD development. Through their implied ability to target mitochondria, disrupt dopamine metabolism, and participate in the formation of oxidants, these toxins initiate a cascade of deleterious events that can cause the progressive degeneration observed in disease [61].

In addition to the prototypical example of MPTP, a host of other compounds that generate oxidative and nitrative stress (defined as the formation of nitric oxide-dependent oxidants) have been found to be harmful to neurons. These agents have been used for the creation of intoxication models in rodents and non-human primates that reproduce some of the neuropathological findings and behavioral symptoms of the human disease. These intoxication models described below are valuable in understanding the role of oxidative mechanisms, mitochondrial dysfunction, and protein aggregation in neuron death and the selective vulnerability of the nigrostriatal pathway.

Mechanistically, MPP+, the active metabolite of MPTP, is selectively taken up by dopaminergic neurons where it is suggested to inhibit complex I of the mitochondrial respiratory chain, inhibit the uptake of dopamine, and participate in oxidation-reduction biochemistry [62]. MPTP administration, widely used in non-human primates and mice, has been shown to replicate many features of PD, including motor phenotype, degeneration of nigral dopaminergic neurons, and formation of α-synuclein positive filamentous proteinaceous inclusions resembling Lewy Bodies [63-66].

The concept that oxidative processes are playing a major role in the demise of the catecholaminergic neurons is reinforced by data documenting that mice over-expressing the antioxidant protein cytosolic SOD1 [67] are protected against MPTP toxicity. Additionally, the contribution of reactive nitrogen species to MPTP-induced neuron injury is revealed by studies in nitric oxide synthase (NOS) deficient animals. MPTP toxicity is attenuated in either iNOS or nNOS deficient mice [68,69] or mice that are treated with nNOS inhibitors [70,71] suggesting that nitric oxide-derived oxidants are participants in the oxidative and nitrative processes that lead to MPTP-induced neurodegeneration.

The herbicide paraquat, a biologically redox active molecule, is a toxin that has deleterious effects on neurons. Paraquat is used in mouse models of neurodegeneration and leads to reduced motor activity, cell death selectively within the dopaminergic neurons of the substantia nigra, and degeneration of the striatal fibers in a dose-dependent way [72,73]. Additionally, systemic administration of paraquat results in upregulation of α-synuclein expression and the formation of aggregates [74], similar to changes that have been reported following administration of MPTP [75]. Overexpression of SOD in cells or mice has been shown to protect against paraquat toxicity, supporting the role of oxidative stress in neuron death [76-79]. Delivery of molecules with SOD/catalase and antioxidant scavenging capacity such as MnTBAP or EUK-189 were shown to have a similar effect [80-82], although recent studies have indicated that this protection against paraquat may be due to antioxidant-independent mechanisms of MnTBAP including prevention of mitochondrial Ca^2+ ^accumulation [83,84].

Rotenone is an insecticide that selectively inhibits mitochondrial complex I. It has been used in rat models to produce a Parkinson-like phenotype including selective degeneration of the dopaminergic neurons of the nigrostriatal region, motor impairment, and fibrillar inclusions [85]. Unlike MPTP, rotenone is highly lipophilic and consequently can enter any cell type [86]. Therefore, rotenone could potentially inhibit mitochondrial complex I throughout the brain. However, rats chronically infused with rotenone develop selective nigral degeneration and α-synuclein positive, Lewy body-like inclusions indicating that dopaminergic cells are somehow exquisitely sensitive to mitochondrial impairment [85]. The molecular details underlying this inherent dopaminergic neuron vulnerability still require further investigation, and will be discussed below.

Finally, 6-hydroxydopamine (6-OHDA), a prototypical oxidative stress toxin used in animal models for over 30 years, mimics PD by causing degeneration of the dopaminergic neurons [87,88]. 6-OHDA is structurally similar to dopamine and norepinephrine and thus can accumulate in catecholaminergic cells. In the presence of oxygen and transition metals it oxidizes into *para*-quinone and hydrogen peroxide, with superoxide (O_2_^.-^) and semi-quinone radicals as intermediate species of the reaction [89]. The generation of reactive species and strong electrophiles attacking nucleophilic groups and inactivating macromolecules have been shown to contribute to neurodegeneration [87,88]. Injection of 6-OHDA in the substantia nigra of rats leads to rapid death of dopaminergic neurons, while injection in the stiatum induces a retrograde degeneration of the neurons in the substantia nigra [90-92].

### Genetic Links

While the majority of PD cases are sporadic, rare instances of genetic heritability have helped to provide further insight into the mechanisms contributing to disease. Currently, thirteen genetic loci, denoted PARK1–13, have been associated with PD [93]. From these loci, six genes have been established as a causative factor of familial PD: α-synuclein (PARK1/4), parkin (PARK2), PINK1 (PARK6), DJ-1 (PARK7), LRRK2 (PARK8), and ATP13A2 (PARK9) [93-95]. ATP13A2 is a lysosomal P-type ATPase that has been associated with a recessive juvenile form of PD [96]. A recent studies highlighted a genetic interaction between ATP13A2 and α-synuclein and showed that ATP13A2 is able to modulate α-synuclein toxicity [97]. However, while ATP13A2's lysosomal location reinforces the importance of autophagic degradation, this review will focus on the other five PD genes that have been most extensively investigated. Each of these five genes (DJ-1, PINK1, Parkin, LRRK2, and α-synculein) has yielded data supporting critical associations with mitochondrial and oxidative processes and protein degradation.

#### DJ-1

Mutations and deletions in the gene encoding DJ-1 have been linked to recessive familial PD. DJ-1 is a mitochondrial-associated protein which has been suggested to function as an antioxidant with peroxidredoxin-like activity [98-100]. Mass spectrometry and other methodologies have indentified Cys106 in DJ-1 as the critical amino acid for DJ-1 mediated protection against oxidative stress as well as for the relocation of DJ-1 to the mitochondria during oxidative stress [101]. Irreversible oxidation of this residue renders the protein incapable of protecting cells from oxidant insults [102].

Support for a role of DJ-1 as a protective antioxidant protein is derived from experiments demonstrating that knockout/knockdown of DJ-1 or expression of DJ-1 with a pathogenic mutation in cells leads to an increased sensitivity to oxidative stress [99,103]. Similarly, increased sensitivity to neurotoxins that generate oxidative stress such as MPTP, rotenone, and paraquat has been documented in DJ-1 deficient drosophila and mice [104-108]. Correspondingly, over-expression of DJ-1 protects against oxidative insults. In dopaminergic cell lines, overexpression of wild type, but not mutant, DJ-1 was able to protect cells from hydrogen peroxide and 6-OHDA challenges, leading to reduced levels of reactive species, protein oxidation, and cell death [109,110]. In animal models, overexpression of wild type but not mutant DJ-1 was protective against dopaminergic neural degeneration in mice exposed to MPTP or rats exposed to 6-OHDA [108,110,111].

#### PINK1

PTEN-induced kinase 1(PINK1) is a mitochondrial associated protein whose loss of function mutations lead to a recessive form of hereditary early onset PD [112]. PINK1 is a putative serine/threonine kinase with an N-terminal mitochondrial targeting sequence [113]. Both endogenous and recombinant PINK1 are localized to the mitochondria in cell culture and a drosophila model [112-114]. Functionally, it is postulated that PINK1 phosphorylates mitochondrial proteins in response to cellular stress and thus protects against mitochondrial dysfunction [112,115]. Additional roles for PINK1 in regulating mitochondrial fusion and fission as well as modulating proteolytic activity through interaction with the serine protease HtrA2 have also been proposed [116-119]. Within the context of disease, lymphoblasts of patients with mutations in PINK1 show increased lipid peroxidation and defects in mitochondrial complex I activity [120,121]. Additionally, abnormal mitochondrial morphology was evident in primary cells derived from patients with two different mutations in PINK1 [120].

PINK1 has been shown to influence cell viability. Knockdown of PINK1 in SH-SY5Y, HeLa, and mouse primary neurons, caused abnormal mitochondrial morphology, compromised mitochondrial function, increased markers of oxidative stress, and ultimately decreased cell viability [120,122]. Additionally, those cells were more vulnerable to challenges by rotenone and the active metabolite of MPTP, MPP+ [120,123,124]. Conversely, overexpression of PINK1 in cell models protected against cell death induced by mitochondrial permeability transition pore opening, oxidative stress, and proteasome inhibitors. Protection of cellular viability was related to the ability of PINK1 to prevent loss of mitochondrial membrane potential, to suppress cytochrome c release from mitochondria, and suppress activation of caspase-3 [112,115,125,126]. Expression of PINK1 with pathogenic mutations, expression of a truncated form of PINK1, or expression of PINK1 lacking the kinase domain eliminated this protective effect [112,115,125,126].

Similar to the cell models, mitochondrial abnormalities and increased sensitivity to stressors have also been documented in PINK1 deficient drosophila [127-130]. This phenotype was able to be rescued by expression of wild type but not mutant PINK1 as well as by expression or administration of SOD-1, further supporting the view that the protective role of PINK1 is related to oxidative processes [128,130].

Interestingly, PINK1 knockout mice do not display generalized mitochondrial defects [131]. However, impaired mitochondrial respiration does occur specifically within the nigrostriatal dopaminergic circuit, and mitochondria isolated from the knockout mice display increased sensitivity to hydrogen peroxide [132]. PINK1 knockout mice also have impaired dopamine release and impaired synaptic plasticity, suggesting a specific role in dopaminergic neuron function [131]. This has important implications for the specificity with which dopaminergic neurons are affected in PD.

#### Parkin

Identification of loss of function mutations in the gene encoding the ubiquitin ligase parkin in autosomal recessive PD indicates that dysfunction of the ubiquitin proteasome system is a contributing factor in the pathogenesis of PD [133-135]. Additionally, recent evidence implicates parkin in mitochondrial function and oxidative processes.

Parkin is localized to the mitochondria of proliferating cells and influences mitochondrial biogenesis [136]. Attempts to examine the effect of parkin modifications on other proteins have included two dimensional gel electrophoresis combined with proteomic analysis in parkin knockout mice, as well a genetic screen for parkin modifiers and the use of cDNA microarrays to characterize transcriptional alterations in parkin deficient drosophila [137-139]. These studies report that parkin modulates expression of proteins involved in the regulation of energy metabolism, such as subunits of pyruvate dehydrogenase, mitochondrial complexes I and IV, and ATP synthase, as well as in proteins involved protection against oxidative stress, such as peroxiredoxin 1, 2, and 6, Hsp70 related proteins, carbonyl reductase, and thioredoxin reductase [137,138]. Drosophila models deficient in parkin or expressing parkin with a pathogenic mutation exhibit mitochondrial dysfunction and alterations in oxidative response components [139,140]. Additionally, parkin deficient drosophila have increased sensitivity to paraquat [141]. In parkin knockout mice, impaired mitochondrial function and decreased antioxidant capacity is accompanied by nigrostriatal defects, synaptic dysfunction, and dopaminergic behavioral deficits [138,142].

Parkin overexpression in cultured cells helped prevent mitochondrial swelling, cytochrome c release, caspase 3 activation, increased reactive species levels, and cell death [143,144]. In a murine model, viral overexpression of parkin was able to inhibit dopaminergic neural loss in mice exposed to MPTP [111]. As an E3 Ubiquitin ligase, parkin levels are upregulated in response to unfolded protein reponse stress induced by the application of the *N*-glycosylation inhibitor tunicamycin or the reducing agent 2-mercaptoethanol [145]. Parkin overexpression correspondingly is able to rescue cells from the unfolded protein response (UPR) induced by various stressors [145]. Additionally, parkin overexpression has been shown to protect cells against proteasomal dysfunction and death induced by mutant α-synuclein [146]

Oxidative modifications may also impact parkin itself. S-nitrosylation, a nitric oxide-derived post-translational modification, of parkin occurs in vitro, in a mouse model of PD, and in the brains of PD patients [147]. S-nitrosylation decreases parkin's ubiquitin E3 ligase activity and its protective function in cells expressing α-synuclein and synphilin-1 that were exposed to a proteasome inhibitor [147,148]. Such consequences provide a mechanism by which parkin's function may be disrupted and thus contribute to disease progression in sporadic PD. S-nitrosylation has also been shown to affect the activity of other proteins relevant to neurodegeneration, including protein-disulfide isomerase (PDI), an ER chaperone [149]. S-nitrosylation inhibits PDI's enzymatic activity, preventing it from promoting the proper folding of proteins during times of cellular stress and preventing PDI's protective effect [149].

Recent studies have provided further support for the role of parkin in oxidative processes by establishing that parkin functions downstream of PINK1 within the same pathway. Drosophila mutants that are deficient in either parkin or PINK1 exhibit similar phenotypes. Transgenic expression of parkin is able to rescue the phenotype of PINK1 deficient flies, although the reverse is not true [127-129]. This downstream relationship is supported by the fact that in PINK1 deficient flies, the level of parkin protein is significantly reduced [128]. Additionally, it has been shown that DJ-1 with a pathogenic mutation is able to associate with parkin, and this association is promoted by oxidative stress [150].

#### Leucine-rich repeat kinase 2

Recently, leucine-rich repeat kinase 2 (LRRK2) has been recognized as a cause of an autosomal dominant late-onset form of familial PD. LRRK2 expression in the brain largely correlates to the nigrostriatal dopaminergic system, although diffuse expression throughout the brain has also been noted, including expression in the cerebral cortex, hippocampus, and cerebellum [151-154]. Within the cell, LRRK2 associates largely with membrane bound structures, including the mitochondria, lysosomes, plasma membrane, synaptic vesicles, golgi apparatus, and endoplasmic reticulum and is likely associated with lipid rafts in these membranes [154-156]. LRRK2 contains putative GTPase, protein kinase, WD40 repeat, and leucine-rich repeat (LRR) domains, but the endogenous function of the protein is still being investigated [157].

In support of the role of mutated LRRK2 in neurodegeneration, expression of LRRK2 with pathogenic mutations in SH-SY5Y cells and primary neurons reduced cell viability [155,158-160]. LRRK2 also affects the ability of the cell to handle oxidative stress. Overexpression of mutant LRRK2 failed to rescue cultured cells from hydrogen peroxide exposure, while expression of wild type LRRK2 successfully attenuated this stress [161]. Additionally, drosophila expressing mutant LRRK2 were significantly more sensitive to paraquat and hydrogen peroxide than flies expressing wild type LRRK2 or deficient in LRRK2 [162]. The magnitude of oxidative damage was lowest in drosophila deficient in LRRK2, while flies expressing the mutant LRRK2 had the highest levels [162]. While these observations support the dominant-negative effect of LRRK2 mutations, it is unclear why wild type LRRK2 is more detrimental than a deficiency of LRRK2. Further studies need to be conducted to fully understand both the normal and pathogenic function of this protein.

#### α-Synuclein

In addition to the discovery that three different autosomal dominant missense mutations in the gene encoding α-synuclein cause early onset, familial PD, wild type α-synuclein has also been identified as one of the primary components of Lewy bodies in sporadic cases [163-167]. α-Synuclein is a soluble, relatively unstructured protein, expressed throughout the central nervous system whose function relates to synaptic vesicular regulation and to chaperone-like activity [168-170]. A hydrophobic region spanning residues 71–82, as well as factors that have not been fully understood, contribute to the orderly assembly of α-synuclein into amyloid fibers that ultimately constitute in part the Lewy bodies and other inclusions [171-173]. α-Synuclein appears to both contribute to mitochondrial dysfunction, oxidative stress, and impaired protein degradation, as well as itself be a target for oxidative modifications that may affect aggregation and neurotoxicity.

In a cell model, overexpression of α-synuclein led to mitochondrial dysfunction and increased levels of reactive species [174]. A similar effect was reported in transgenic mice expressing α-synuclein with the A53T pathogenic mutation. These mice developed mitochondrial degeneration and cell death [175]. α-Synuclein additionally appears to sensitize mice to mitochondrial toxins. Transgenic mice expressing mutant α-synuclein had increased neural degeneration, mitochondrial abnormalities, α-synuclein aggregation, and levels of oxidative and nitrative modifications after exposure to challenges including MPTP, paraquat and maneb [176-179]. Importantly, mice that lack α-synuclein are protected against MPTP toxicity [180-182]. Recent evidence has also shown that α-synuclein accumulates within mitochondria due to an N-terminal targeting sequence, leading to impaired mitochondrial complex I activity and increased production of reactive species [183]. Significantly more α-synuclein was accumulated in mitochondria isolated from the substantia nigra and striatum of patients with sporadic PD than from controls [183].

α-Synuclein may also play a role in disease through its effects on protein degradation. It has been suggested that α-synuclein may initiate UPS inhibition, as it has been shown to disrupt the proteasome *in vitro*, an effect that is enhanced by the pathogenic α-synuclein mutations [146,184-186]. The mechanisms underlying this inhibition are not fully understood, though possibilities include binding of α-synuclein to a subunit of the proteasome, blockage of the proteasome by aggregated proteins, or potentially an unknown downstream mechanism. Additionally, α-synuclein may play a role in autophagy. *In vitro *studies have shown α-synuclein is preferentially degraded by CMA [187]. However pathogenic mutations of synuclein or modification by oxidized dopamine cause α-synuclein to bind strongly to the lysosomal CMA receptor. This blocks the uptake and degradation of α-synuclein and other CMA substrates [55,187]. Downstream effects of this disruption may explain how α-synuclein mutations are able to induce cell death – α-synuclein induced impaired CMA degradation of myocyte enhancer factor 2D (MEF2D), a transcription factor required for neuronal survival, resulting in the cytosolic accumulation of MEF2D that bound poorly to DNA, causing an overall decrease in MEF2D function [188].

While α-synuclein can modulate mitochondrial function, oxidative challenges, and protein degradation machinery, oxidation and nitration also appear to modify α-synuclein directly and consequently affect its aggregation. α-Synuclein nitrated on tyrosine residues has been identified in the detergent-insoluble fraction of the brains of PD patients, suggesting that this modification may induce the aggregation of this protein or that aggregated forms of the protein are selectively modified by nitrating oxidants [189]. In cell, mouse, and non-human primate models, treatment with MPTP has been shown to increase oxidative modifications and aggregation of α-synuclein [64,75,190]. Treatment of cells or rats with rotenone and mice with paraquat similarly increased α-synuclein aggregation and inclusion formation and cellular dysfunction [74,85,191].

Collectively, these findings led to biochemical examination of the effect of oxidative or nitrative modification on α-synuclein. Fibrillar α-syncuclein aggregates with a perinuclear localization were formed in cells expressing α-syncuclein upon kinetically controlled exposure to nitric oxide and superoxide [192]. Studies with purified protein revealed that tyrosine nitration effects the ability of α-synuclein to bind to lipid vesicles and slows the rate of degradation by the 20S proteasome and calpain-I [193]. Nitration of α-synuclein monomers and dimers is able to accelerate the rate of fibril formation through the recruitment of non-nitrated α-synuclein, but nitration of oligomers inhibits fibril formation [193-195]. In addition to nitration, exposure of α-synuclein to nitrating oxidants also results in the formation of highly stable *o*, *o'*-dityrosine cross linked dimers and oligomers [196]. *o*, *o'*-Dityrosine cross linking was found to stabilize pre-formed fibrils, which significantly accelerate the formation of fibrilar aggregates. Site-directed mutation of the four tyrosine residues in α-synuclein discerned that the tyrosine residues are essential for crosslinking and stabilization in response to nitrative insults. [196]. However, oxidative modifications also are able to affect α-synuclein and invoke crosslinking and stable fibril formation independent of tyrosine residues [197]. The C-terminal of α-synuclein has been found to be critical for oligomerization of α-synuclein into detergent insoluble species in response to oxidation by copper and hydrogen peroxide [198].

Due to the regional specificity of pathology in PD patients, the effect of dopamine on α-synuclein has also been investigated. During a chemical compound library screen for molecules that would inhibit formation of α-synuclein fibrils, Lansbury and coworkers discovered that the neurotransmitter dopamine inhibits the formation of α-synuclein fibrils [199]. The interaction of dopamine with α-synuclein appeared to arrest the process of fibril formation at a stage of oligomeric species [199]. We have extended these observations to indicate that dopamine oxidation is essential for this kinetic arrest of α-synuclein oligomers [200]. Since dopamine oxidation generates reactive species and strong electrophiles, mutational analysis of putative amino acid targets in α-synuclein that could be modified by this oxidation was explored [200]. Examination of sites such as the three methionine residues and histidine 50 revealed that covalent modification of these amino acids was not responsible for the effects of oxidized dopamine [200]. The data indicated that the interaction of oxidized dopamine with α-synuclein is directed, not towards a single amino acid, but rather five amino acid residues: tyrosine-glutamate-methionine-proline-serine (YEMPS) in position 125–129 in the C-terminus of the protein [200,201]. Recent studies have confirmed these findings and also indicated that the glutamate 83 residue also participates in stabilizing the interaction of oxidized dopamine with the YEMPS region [202]. The in vitro data has been confirmed in cellular model systems that express A53T α-synuclein or A53T α-synuclein with all 5 amino acids 125–129 mutated, establishing the importance of this C terminal region in the stabilization of α-synuclein oligomers in the presence of oxidized dopamine [201,203]. The decrease in catecholamine levels that has been described as an early even in PD pathogenesis [204] may then allow the formation of insoluble α-synuclein aggregates later in disease [203]. Additionally, α-synuclein modified by oxidized dopamine may have deleterious effects on cellular function, indicating that aggregation may not be a necessary prerequisite for cell death. α-Synuclein modified by oxidized dopamine has been shown to block CMA by binding strongly to the L2A receptor and blocking the uptake of itself and other substrates [55]. Oligomeric α-synuclein was shown to bind to the lysosomal membrane but was unable to be unfolded or taken up into the lysosomes [55]. Furthermore, α-synuclein modified by oxidized dopamine was able to decrease neuronal viability to a degree similar to the effect of L2A RNAi [55]. Therefore α-synuclein may serve as both a modulator and a target of oxidative and nitrative modifications.

### Endogenous Factors

In addition to evidence from genetic and environmental risks, the two endogenous factors of aging and dopamine oxidation have implicated oxidative modifications, mitochondrial dysfunction, and impaired protein degradation in PD.

#### Aging

In PD, the most significant risk factor for developing disease is age. The accumulation of proteins altered by oxidative modifications has been shown to increase with age, which correlates with the late-onset of neurodegenerative pathology [205,206]. Examination of cultured human fibroblasts, human brain tissue, as well as tissues from other organisms have shown that in elderly individuals, approximately one third of proteins have been oxidatively modified [206-208]. This increase is not linear but instead occurs as an initial gradual rise that magnifies several fold in late age [6,206-208]. Oxidative modifications most likely accumulate with age due to a combination of increased production of reactive species, decreased antioxidant function, and impaired ability to repair or remove the modified proteins.

Dysfunctional clearance has been largely supported by findings that the activities of the UPS, macroautophagy and CMA decline with age, consequently diminishing the ability of the cell to clear modified proteins or protect itself from damaging free radicals [47,209-216]. Due to impaired degradation, proteins with oxidative modifications accumulate in the cell, increasing their propensity for aggregation [47,216]. Additionally, once the activity of these degradation pathways is diminished, a feed-forward effect on oxidative damage may result. Sullivan et al. found that proteasomal inhibition increased mitochondrial reactive species generation and decreased mitochondrial complex I and II activity [217]. Therefore, inhibition of the proteasome and autophagy pathways may be further contributing to oxidative damage.

#### Dopamine Oxidation

The characteristic topology of cell loss that is revealed from neuropathological studies of PD brains, with the relatively selective vulnerability of the ventrolateral and caudal regions of the substantia nigra pars compacta, can provide useful clues on the etiology of the disease. In particular, it has been postulated that the oxidative environment of dopaminergic neurons might be a key component in the pathogenesis of PD. Typically, dopamine is rapidly sequestered within vesicles by the vesicular monoamine transporter, where the acidic pH significantly delays the oxidation of dopamine. However, an oxidative environment can be created if dopamine remains in the cytosol, where it can oxidize at physiological pH to generate reactive ortho-quinones, aminochromes, as well as superoxide and hydrogen peroxide [218,219]. Excessive cytosolic oxidation of catechols has been shown to be neurotoxic in cell culture and rodent models [220-222]. However, it is unclear whether intracellular oxidation of dopamine is able to significantly contribute to the neuron injury.

The gradual accumulation of oxidized dopamine that occurs in normal aging does not appear sufficient to induce neuronal death. However, a consequence of the accrual of oxidized dopamine is the formation of neuromelanin. Neuromelanin, the substance that gives the dopaminergic neurons of the substantia nigra their characteristic dark appearance, is a polymer of oxidized and subsequently heterocyclized dopamine. It has been proposed that the polymer is sequestered within the neurons to form a new cellular organelle of unknown function [223]. In that capacity it has been hypothesized that the neuromelanin polymer might be neuroprotective by further chelating toxins and transition metals such as iron and manganese [223-226]. Since divalent redox capable metals such as iron participate in catalytic reactions with hydrogen peroxide to generate potent oxidizing species, such a role would be crucial to protecting neurons. Efforts to limit the availability of iron have been found to protect neurons from injury and death [227-230].

Alternatively, other studies have revealed a correlation in PD brains between cell loss and the presence of neuromelanin, which suggests that the neuromelanin-pigmented subpopulation of dopaminergic neurons are more vulnerable in disease [231]. Another interesting but unexplored observation is the co-localization of the characteristic protein inclusions (Lewy bodies) in close proximity to neuromelanin in human post mortem PD brains [232,233]. It is possible that the synthesis of neuromelanin, which requires the oxidation of dopamine and the formation of oxidants and electrophiles, promotes the formation of protein aggregates by oxidizing proteins, providing a scaffold for protein filament assembly, or both. In support of its role as a scaffold for aggregation, the melanosome has been shown to be crucial for the assembly of the non-pathogenic natively amyloidogenic protein Pmel17 [234]. Additionally, the melanosome precursor itself assembles into amyloid-like fibrils that may promote the association and assembly of other amyloidogenic proteins [235]. Aggregation may also been promoted by the raft like lipid component of neuromelanin, as hydrophobic interactions would bring macromolecules in close proximity [235,236]. Another interesting observation is that the presence of neuromelanin in dopaminergic neurons is unique to primates, which may explain inconsistencies in the attempts to recapitulate disease in rodent models [237-240].

## Conclusion

Examining the "Bermuda triangle" in which dopamine neurons are lost, oxidative modifications, mitochondrial dysfunction, and impaired protein degradation appear to be three interrelated molecular pathways responsible for the pathogenesis of both sporadic and familial PD (Figure 1). Evidence from environmental, genetic, and endogenous factors highlights the interplay of these three mechanisms as the common detrimental denominators inducing neuronal death. Not only do these three processes have clear impacts on cellular viability, but their participation explains other characteristic features of disease, such as the presence of oxidized proteins, inclusions, increased prevalence with late age, and dopaminergic regional selectivity. Together, through their effects on cellular homeostasis and their interactions with one another, oxidative stress, mitochondrial dysfunction, and impaired protein degradation provide the final impetus with which insult to neurons is transformed into neurodegenerative disease.

Currently, treatment for PD is focused merely on alleviating symptoms. As research progresses towards a better understanding of the molecular mechanisms underlying disease, hopefully a more effective therapy can ultimately be designed. Current trials to deliver compounds that can restore mitochondrial function and reduce oxidative burden will be informative and not only improve therapeutic treatment of PD but also provide vital results to guide future studies investigating the molecular mechanisms of neurodegeneration.

## Abbreviations

PD: Parkinson's Disease; UPS: Ubiquitin Proteasome System; CMA: Chaperone Mediated Autophagy; MPTP: 1-methyl-4-phenyl-1,2,3,6-tetrahydropyridine; 6-OHDA: 6-Hydroxy Dopamine; PINK1: PTEN-Induced Kinase 1; LRRK2: Leucine-Rich Repeat Kinase 2; SOD: Superoxide dismutase; NOS: Nitric oxide synthase.

## Competing interests

The authors declare that they have no competing interests.

## Authors' contributions

KAM, ET, and HI wrote the manuscript.
